# Chronic Nicotine Exposure *In Vivo* and *In Vitro* Inhibits Vitamin B1 (Thiamin) Uptake by Pancreatic Acinar Cells

**DOI:** 10.1371/journal.pone.0143575

**Published:** 2015-12-03

**Authors:** Padmanabhan Srinivasan, Edwin C. Thrower, Gopalakrishnan Loganathan, A. N. Balamurugan, Veedamali S. Subramanian, Fred S. Gorelick, Hamid M. Said

**Affiliations:** 1 Department of Medical Research, VA Medical Center, Long Beach, California, United States of America; 2 Departments of Medicine and Physiology/Biophysics, University of California Irvine, Irvine, California, United States of America; 3 Clinical Islet Cell Laboratory, Department of Surgery, Cardiovascular Innovation Institute, University of Louisville, Louisville, Kentucky, United States of America; 4 Section of Digestive Diseases, Department of Internal Medicine, School of Medicine, Yale University, New Haven, Connecticut, United States of America; 5 Section of Digestive Diseases, Department of Internal Medicine, Yale University, New Haven, Connecticut; 6 Veterans Affairs Healthcare System, West Haven, Connecticut, United States of America; University of Szeged, HUNGARY

## Abstract

Thiamin (vitamin B1), a member of the water-soluble family of vitamins, is essential for normal cellular functions; its deficiency results in oxidative stress and mitochondrial dysfunction. Pancreatic acinar cells (PAC) obtain thiamin from the circulation using a specific carrier-mediated process mediated by both thiamin transporters -1 and -2 (THTR-1 and THTR-2; encoded by the *SLC19A2* and *SLC19A3* genes, respectively). The aim of the current study was to examine the effect of chronic exposure of mouse PAC *in vivo* and human PAC *in vitro* to nicotine (a major component of cigarette smoke that has been implicated in pancreatic diseases) on thiamin uptake and to delineate the mechanism involved. The results showed that chronic exposure of mice to nicotine significantly inhibits thiamin uptake in murine PAC, and that this inhibition is associated with a marked decrease in expression of THTR-1 and THTR-2 at the protein, mRNA and hnRNAs level. Furthermore, expression of the important thiamin-metabolizing enzyme, thiamin pyrophosphokinase (TPKase), was significantly reduced in PAC of mice exposed to nicotine. Similarly, chronic exposure of cultured human PAC to nicotine (0.5 μM, 48 h) significantly inhibited thiamin uptake, which was also associated with a decrease in expression of THTR-1 and THTR-2 proteins and mRNAs. This study demonstrates that chronic exposure of PAC to nicotine impairs the physiology and the molecular biology of the thiamin uptake process. Furthermore, the study suggests that the effect is, in part, mediated through transcriptional mechanism(s) affecting the *SLC19A2* and *SLC19A3* genes.

## Introduction

Thiamin (vitamin B1), a member of the water-soluble family of vitamins, is required for the normal health and function of pancreatic acinar cells (PAC). The vitamin acts as a cofactor for enzymes like transketolase, pyruvate dehydrogenase, alpha-ketoglutarate dehydrogenase and branched chain ketoacid dehydrogenase that are involved in a variety of cellular metabolic reactions related to energy metabolism. Thiamin also plays a key role in the reduction of cellular oxidative stress and in maintaining mitochondrial health and function. Deficiency of thiamin is detrimental for normal cell physiology and leads to impairment of oxidative energy metabolism (acute energy failure) predisposing the cells to oxidative stress [1, 2]; it further causes mitochondrial dysfunction [3].

The pancreas, with its important endocrine and exocrine functions, plays critical roles in normal health and physiology. Although PAC lack the ability for *de novo* synthesis of thiamin, they maintain it at high levels [4], by extracting it from circulation using specific plasma membrane transporters. Thiamin deficiency leads to a dramatic reduction in pancreatic content of digestive enzymes [5] and to inadequate insulin secretion [6, 7], thus, affecting both the exocrine and endocrine functions of this organ.

We have previously elucidated the mechanism of thiamin uptake by PAC and showed that cellular thiamin uptake is mediated by a specific and regulated carrier-mediated process [8, 9]. We have also utilized *SLC19A2* and *SLC19A3* knockout mouse models to show the involvement of both thiamin transporters THTR-1 and THTR-2 in cellular thiamin uptake [9]. Pancreatic health and physiology are affected by a number of environmental factors. Many experimental and epidemiological studies have linked cigarette smoke (CS) to injury/disease of the pancreas [10–15]; indeed, exposure to CS leads to significant functional and pathological changes in the exocrine pancreas [13, 16, 17]. The effect of CS exposure on cell physiology appears to be multifactorial and includes differential effects on gene expression and oxidative stress, and causes mitochondrial dysfunction [18–22]. Nicotine is a major component of CS and has been extensively studied because of its addictive properties. Although it is not a carcinogen itself, nicotine is a risk factor for the induction/development of pancreatic inflammation and pancreatic cancer [11, 13, 23–26]. Nicotine is known to accumulate in the pancreas [27], and has been implicated in the production of free radicals that lead to oxidative stress and consequently pancreatic injury [28]. Furthermore, animal studies have shown that nicotine exposure induces changes in the pancreas that are similar to those seen in experimental models of pancreatitis [29]. The effect(s) of nicotine on pancreatic physiology appears to be mediated mainly by the nicotinic acetylcholine receptor and elevated levels of intracellular calcium [30]. Nothing is known about the influence of nicotine on the physiology of thiamin uptake by PAC. An essential micronutrient such as thiamin plays critical roles in energy metabolism, mitochondrial function and reduction of cellular oxidative stress; a deleterious effect of nicotine on pancreatic thiamin uptake could contribute to the observed negative impact of CS on the health of PAC [1–3, 31].

In this study we examined the effect of chronic exposure of PAC to nicotine *in vivo* and *in vitro* on thiamin uptake. We used *SLC19A2* and *SLC19A3* transgenic mice previously generated and characterized in our laboratory [32, 33] as an *in vivo* model. Cultured human primary PAC were used for *in vitro* studies. The human PAC were isolated and maintained under culture conditions [34] and were used after 3 days in culture (4 days from isolation). This time period was shown to have minimal effect on cellular morphology and no effect on cell differentiation [34]. Collectively, the results show that nicotine inhibits thiamin uptake by PAC and that this inhibition is mediated, at least in part, at the level of transcription of the thiamin transporter genes.

## Materials and Methods

### Materials

[^3^H]-Thiamin (specific activity 20 Ci/mmol) with a radiochemical purity > 99% was purchased from American Radiolabeled Chemicals (St. Louis, MO). Nitrocellulose filters (0.45-μm pore size) were from Millipore (Fisher Scientific). All other chemicals including unlabelled thiamin and molecular biology reagents were of analytical grade obtained from commercial vendors. Oligonucleotide primers were synthesized by Sigma Genosys (Sigma, Woodland, TX). THTR-1, THTR-2 and β-actin antibodies were purchased from Santa Cruz Biotechnology (Santa Cruz, CA) and mice TPKase rabbit polyclonal antibodies were purchased from Proteintech (Chicago, IL).

### Chronic exposure of mice to nicotine and thiamin uptake by freshly isolated mouse PAC

Transgenic mice carrying the full-length human *SLC19A2* (-2,250 to -36) and *SLC19A3* (-1,957 to +59) promoters fused to the firefly luciferase reporter gene used in this study were previously generated and characterized as described [32, 33]. The animal experimental protocol was approved by the Animal Use Committee of the Long Beach Veterans Affairs Medical Centre. The mice received nicotine in the drinking water at a concentration of 0.77 mM for 4 weeks (a dosage that achieves the same systemic levels of nicotine as that found in smokers; [35]) as described previously [35]. Control animals received regular (no nicotine) water. After 4 weeks the mice were euthanized and the pancreas was removed and primary PAC were isolated by a collagenase type-V (Sigma, St. Louis, MO) digestion method as described previously [36–38]. Freshly isolated PAC were used for uptake analysis on the day of isolation with a portion stored at −80°C for protein, mRNA expression and firefly luciferase analyses. Cells were suspended in Krebs-Ringer buffer (in mM: 133 NaCl, 4.93 KCl, 1.23 MgSO_4_, 0.85 CaCl_2_, 5 glucose, 5 glutamine, 10 HEPES, and 10 MES; pH 7.2; 37°C) labelled and unlabelled thiamin was added at the onset of incubation, and the reaction was terminated after 7 min (initial rate; [8]). A rapid filtration technique was used in uptake investigations as described previously [8]. Carrier-mediated thiamin uptake was determined by subtracting ^3^H-thiamin uptake in the presence of 1 mM of unlabeled thiamin from that in its absence (i. e., from total uptake). Protein concentrations were determined using a Bio-Rad Dc protein assay kit.

### Human pancreatic acinar cell Isolation

PAC were the byproduct of the human islet cell isolation process from de-identified organ donors. Collected human pancreas was digested to obtain islet and acinar cells. Briefly, after informed consent had been obtained, pancreata of subjects (15–65 years old) were removed from brain-dead donors as part of multi-organ procurement. The pancreata were transported either in histidine-tryptophan-ketoglutarate solution or in cold University of Wisconsin solution [39, 40]. On arrival at the laboratory, the pancreas was trimmed, cannulated, and distended with collagenase and neutral protease [41]. After ductal perfusion of the enzyme, the pancreas was digested using a modified Ricordi’s semi-automated method [42]. The dissociated islet and acinar cells were then separated by continuous iodixanol (OptiPrep^™^, Axis-Shield, Oslo, Norway) density gradient on a COBE-2991 cell processor. The sedimented acinar cells were collected from the COBE bag. The collected acinar cells were washed and dispersed in CMRL-1066 (Connaught Medical Research Laboratories) supplemented medium (Mediatech Inc; Manassas, VA) [43].

### Assessment of viability of the isolated human PAC

The rapid fluorescein diacetate/propidium iodide method was used to evaluate the viability of acinar cells after isolation. This assay differentiates viable and nonviable cells by simultaneous staining. The inclusion and exclusion dyes used were fluorescein diacetate and propidium iodide [44]. Individual cells were observed through the fluorescent microscope and scored individually to estimate overall cell viability as a percentage of the preparation. The purity of PAC was ≈ 95% (islet purity was assessed by ditizone staining [45] which provided an indirect measure of acinar cell purity) and the viability was > 90%. The viability was also assessed on the day of use of the cultured PAC for transport investigations using the trypan blue exclusion method and found to be > 80%. Finally, morphology of the human acinar cells was similar to that described previously [34].

### Chronic exposure of human pancreatic acinar cells to nicotine and uptake studies

Human primary PAC were cultured in Ham’s F-12K media with 10% FCS, 5% BSA, 10 ng/ml of epidermal growth factor (EGF) and 0.1 mg/ml soybean trypsin inhibitor as described [34]. After 24 h the cells exhibited attaching and spreading and formed a monolayer. The viability was > 80% (tryphan blue) during the entire culture period (3 days), a value similar to that reported by others [34]. After 24 h of culturing in the media described above, cells were exposed to nicotine (0.5 μM) for 48 h as described [46, 47]. The nicotine concentration used corresponded to that seen in the blood of smokers [48]. The total time between isolation and use of the cells was about 4 days. The nicotine exposed and control human PAC were suspended in Krebs-Ringer buffer for thiamin uptake studies. Labelled and unlabelled thiamin was added at the onset of incubation, and the reaction was terminated after 7 min with addition of cold Krebs-Ringer buffer and a rapid filtration technique was employed for the uptake investigations as described previously [8]. Carrier -mediated thiamin uptake was measured by subtracting ^3^H-thiamin uptake in the presence of 1 mM of unlabeled thiamin from that in its absence (i. e., form total uptake). Protein concentrations were determined using a Bio-Rad Dc protein assay kit.

### Western Blot Analysis

Western blot analysis was performed using 60 μg protein from whole cell lysate prepared from mouse and human PAC chronically exposed to nicotine and their respective controls. Briefly, PAC were suspended in 200 μl of RIPA buffer (Sigma) supplemented with protease inhibitor cocktail (Roche) and the proteins were resolved on premade 4–12% Bis-Tris minigel (Invitrogen). After electrophoresis, proteins were transferred onto immobilon polyvinylidene difluoride membrane (Fisher Scientific) and blocked with Odyssey blocking solution (LI-COR Bioscience, Lincoln, NE). The membranes were incubated overnight either with THTR-1 or THTR-2-specific (1:200 dilution) polyclonal goat antibody along with β-actin (1:3,000 dilution) monoclonal antibody for mice. Mice TPKase was detected using TPKase specific rabbit polyclonal antibodies. For human PAC protein, the membrane was incubated with THTR-1 polyclonal goat antibody, or THTR-2 along with β-actin (1:3,000 dilution) monoclonal antibody. The THTR-1, THTR-2, and β-actin immunoreactive bands were detected by using donkey anti-goat IRDye-800 (for mice THTR-1 and THTR-2) goat anti-rabbit IRDye-800 (for human THTR-2, mice TPKase) and anti-mouse IRDye-680 (for β-actin) (LI-COR Bioscience) secondary antibodies (1:30,000 dilution). Odyssey infrared imaging system (LI-COR Bioscience) was used to detect the signals and quantified using LI-COR software and normalized to β-actin as an internal control.

### Real-Time PCR Analysis

Total RNA (2 μg) was isolated from primary mouse/human PAC and treated with DNase I (Invitrogen) and then subjected to cDNA synthesis using iScript cDNA synthesis kit (Bio-Rad, Hercules, CA). The mice THTR-1, THTR-2, TPKase mice and ARPO mRNA/ heterogeneous nuclear RNA (hnRNA) and human THTR-1, THTR-2 and ARPO mRNA were PCR amplified using specific primers (Table 1) for quantitative PCR studies. Quantitative PCR conditions were the same as described [49]. The data were normalized to ARPO for mice and β-actin for human and then values were calculated by a relative relationship method [50].

**Table 1:** Primers used for amplifying coding region of the respective genes by quantitative PCR.

Gene Name	Forward and Reverse Primers (5′-3′)
Mice quantitative PCR primers	
THTR-1	GTTCCTCACGCCCTACCTTC; GCATGAACCACGTCACAATC
THTR-2	TCATGCAAACAGCTGAGTTCT; ACTCCGACAGTAGCTGCTCA
TPKase	CTCCTGACCAAGACCACA; TGATGTGAGTGGCTTGGA
ARPO	GCTGAACATCTCCCCCTTCTC; ATATCCTCATCTGATTCCTCC
hnRNA-THTR-1	CCCTCTGAAGTCCGTCTCT; ACAGCCCTCAAAAACACCT
hnRNA-THTR-2	CCTCCCTTCCTGTCTTTTC; TTTTCATTGCTGTGGTTGG
hnRNA-ARPO	GGCATCTTCAGTTGTTCC; TTAGACACAGCCCCCAC
Human quantitative PCR primers	
THTR-1	GCCAGACCGTCTCCTTGTA; TAGAGAGGGCCCACCACAC
THTR-2	TTCCTGGATTTACCCCACTG; GTATGTCCAAACGGGGAAGA
β-actin	CATCCTGCGTCTGGACCT; TAATGTCACGCACGATTTCC

### Luciferase assay in transgenic mice

For luciferase assays, mouse PAC were homogenized in ice-cold passive lysis buffer (Promega), and centrifuged to pellet debris (25,000 g, 10 min). Levels of firefly luciferase activity in homogenates were assayed using a Luciferase Assay system (Promega). Luciferase activity was normalized to the total protein concentration of each sample.

### Statistical Analysis

Uptake data with mouse and human PAC presented in this paper are mean ± SE of at least 3 independent experiments (different mice/subjects) and are expressed as a percentage relative to simultaneously performed controls. Carrier-mediated thiamin uptake was determined by subtracting ^3^H-thiamin uptake in the presence of 1 mM of unlabeled thiamin from that in its absence. Protein, mRNA, hnRNA and luciferase activity determinations were performed on at least three sets of samples prepared at different occasions. The Student's *t*-test was used for statistical analysis, and *P* < 0.05 was considered statistically significant.

## Results

### Effect of chronic exposure of transgenic mice carrying the human *SLC19A2* and *SLC19A3* promoters to nicotine on thiamin uptake by freshly isolated PAC: *in vivo* exposure studies

**Fig 1:**
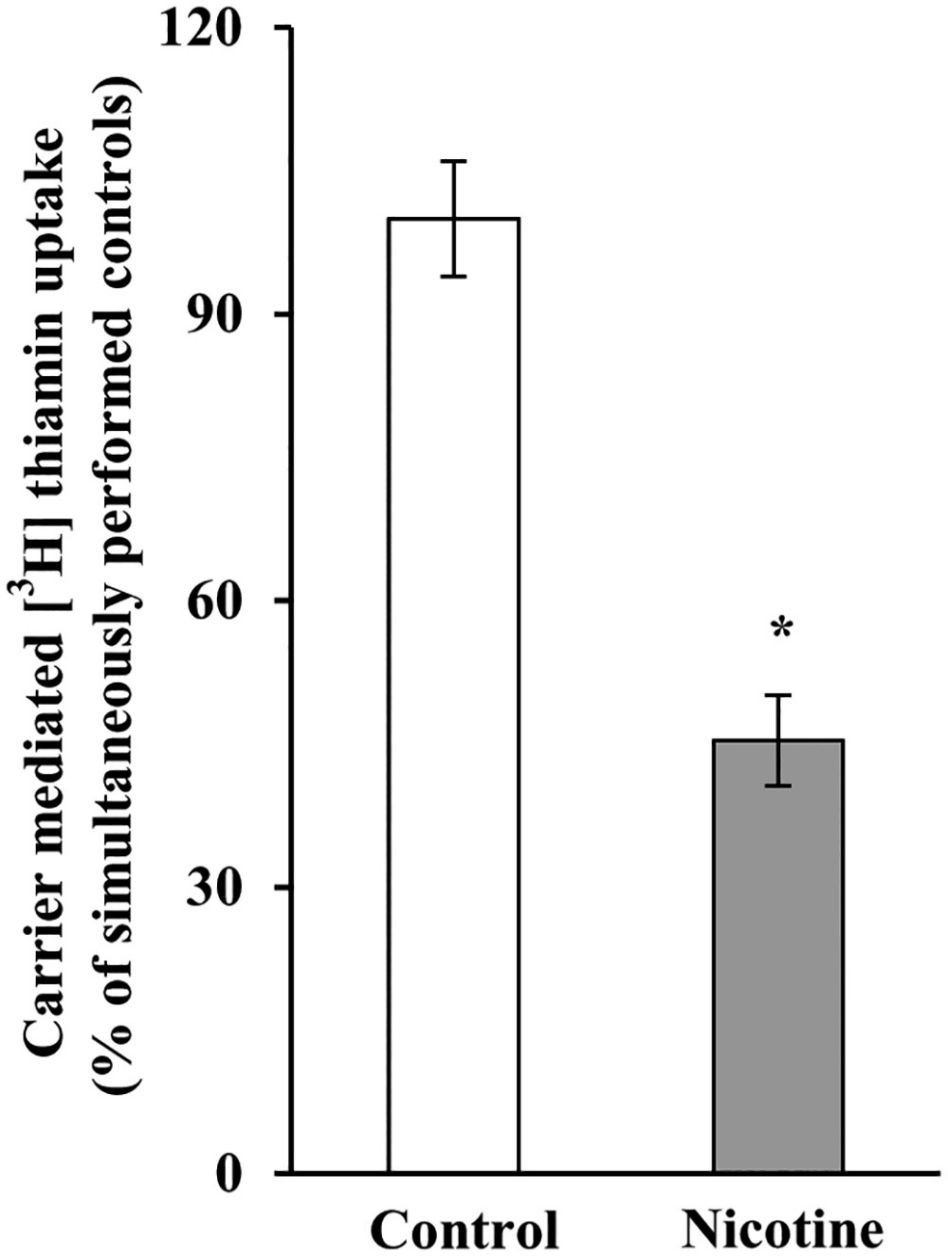
Chronic exposure of mouse primary PAC to nicotine decreases the carrier-mediated [^3^H]thiamin uptake. Primary PAC were isolated from mice exposed to nicotine and their controls. Carrier -mediated [^3^H] thiamin uptake was determined as described in “Materials and Methods”. Data are means ± SE of at least three separate uptake determinations from multiple sets of mice. *P < 0.01.

**Fig 2:**
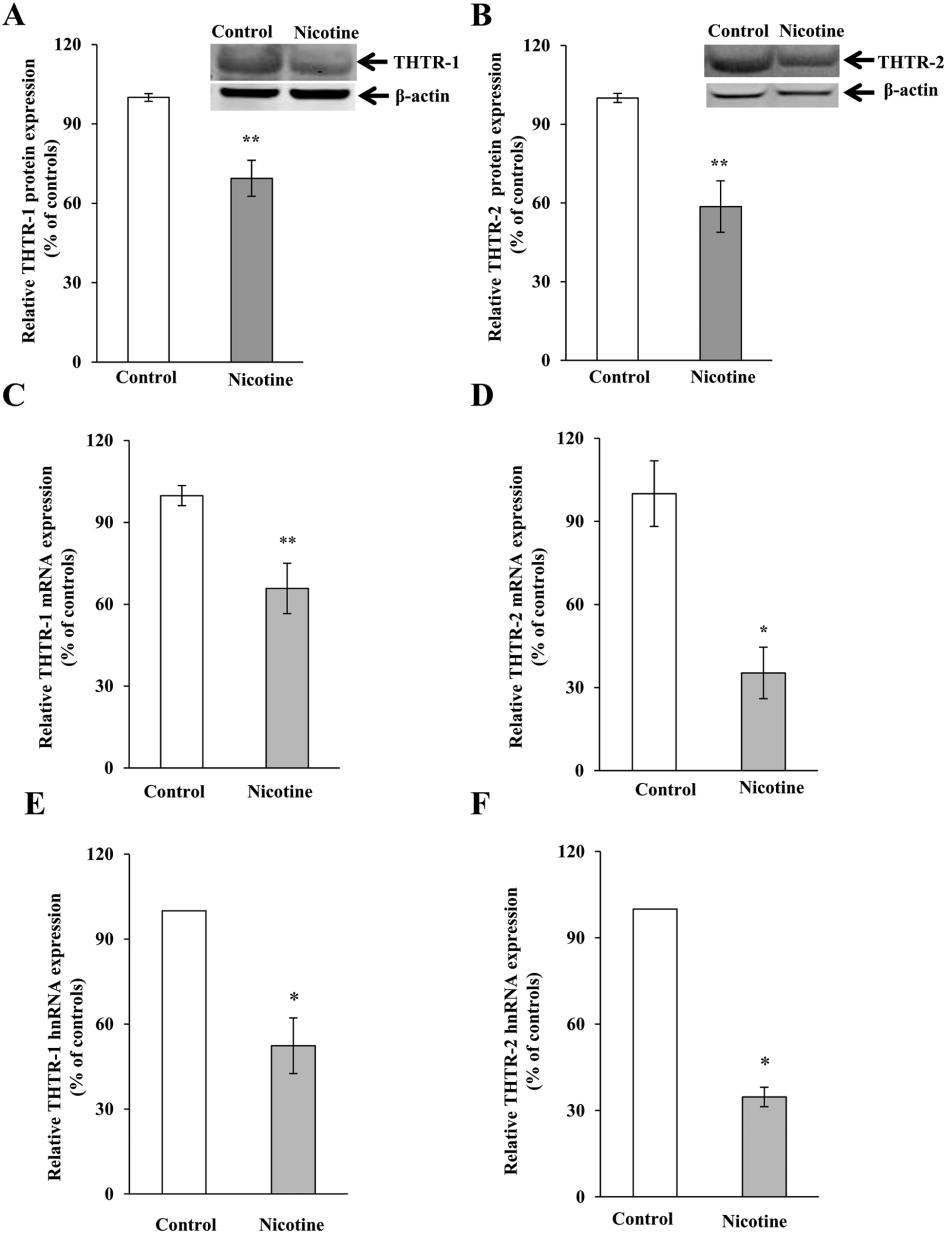
Chronic exposure of mouse primary PAC to nicotine decreases the levels of THTR-1 and THTR-2 proteins (A and B), mRNA (C and D) and hnRNA (E and F). Western blotting was performed on whole cell proteins (60 μg) isolated from chronic nicotine-exposed mice and their controls. Real-time PCR was performed using mice THTR-1 and THTR-2 gene-specific primers. Data are mean ± SE from separate sets of samples from multiple mice and were normalized relative to ARPO and calculated by the relative relationship. *P < 0.01, **P < 0.05.

**Fig 3:**
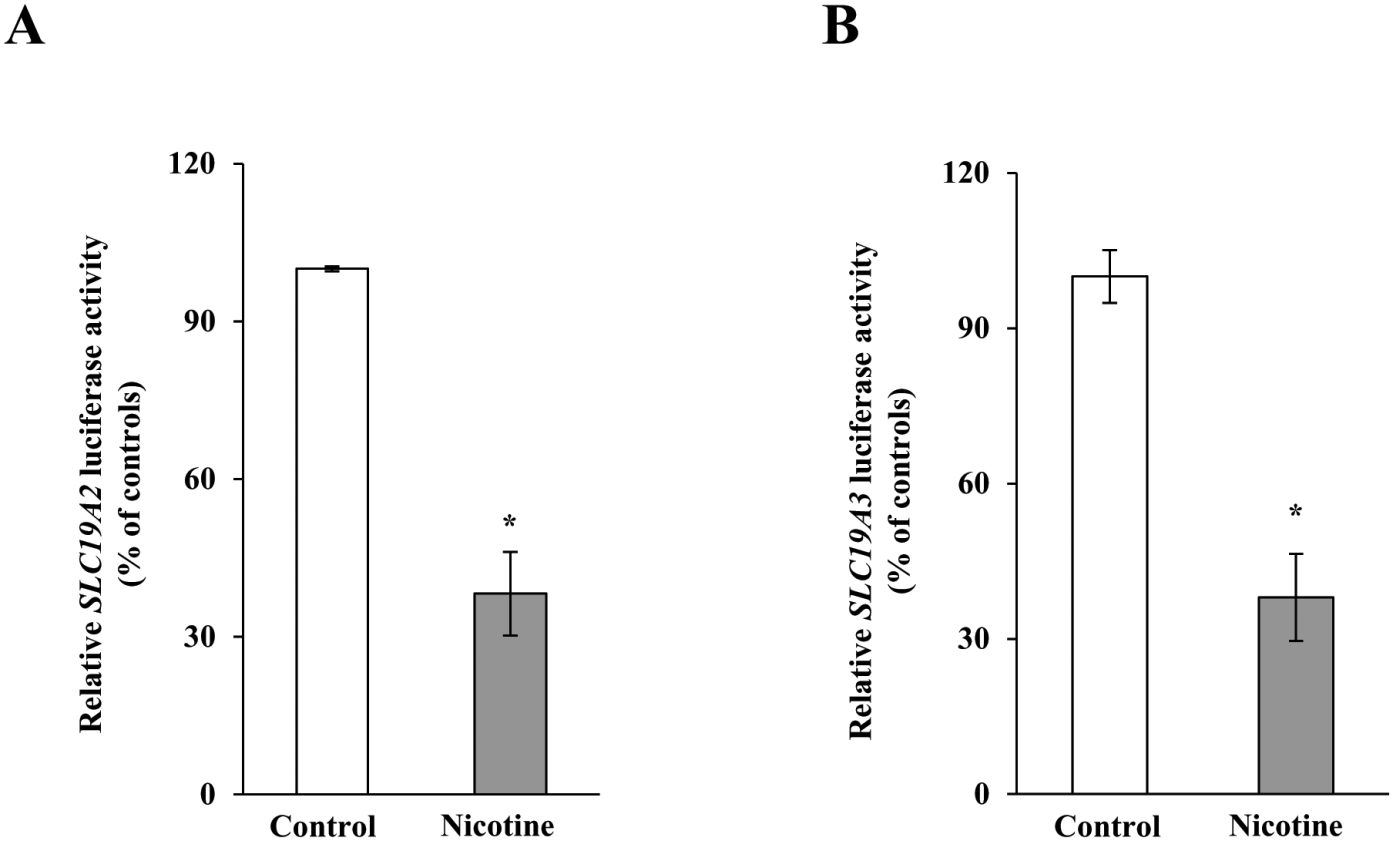
Chronic exposure to nicotine decreases the activity of *SLC19A2* and *SLC19A3* promoters in transgenic mice carrying human *SLC19A2* (A) and *SLC19A3* (B) promoters fused to luciferase. The activities of the promoters were determined as described in “Materials and Methods” and are presented in percentage relative to their controls. Data are mean ± SE of at least three independent experiments from multiple sets of mice. *P < 0.01.

We investigated the effect of chronic exposure of mice to nicotine on the physiological/molecular aspects of thiamin uptake in transgenic mice carrying the *SLC19A2* and *SLC19A3* promoters (fused to the Firefly luciferase reporter gene). Nicotine (0.77 mM) was administered in the drinking water for 4 weeks as described [35]. The results showed a substantial (*P <* 0.01) inhibition in thiamin uptake by freshly isolated PAC (Fig 1). We further examined, by means of Western blotting and quantitative PCR, the effect of chronic exposure of mice to nicotine on the level of expression of the mouse THTR-1 and THTR-2 proteins and mRNAs. The results showed a significant reduction in the expression of these two transporters at the protein (*P <* 0.05), mRNA (*P <* 0.01) and hnRNA (*P <* 0.01) levels in PAC from nicotine-treated mice versus the controls (Fig 2). Finally, we observed a substantial (*P <* 0.01) decrease in the activity of the *SLC19A2* and *SLC19A3* promoters in PAC from nicotine-treated transgenic animals compared to their control transgenic mice (Fig 3).

**Fig 4:**
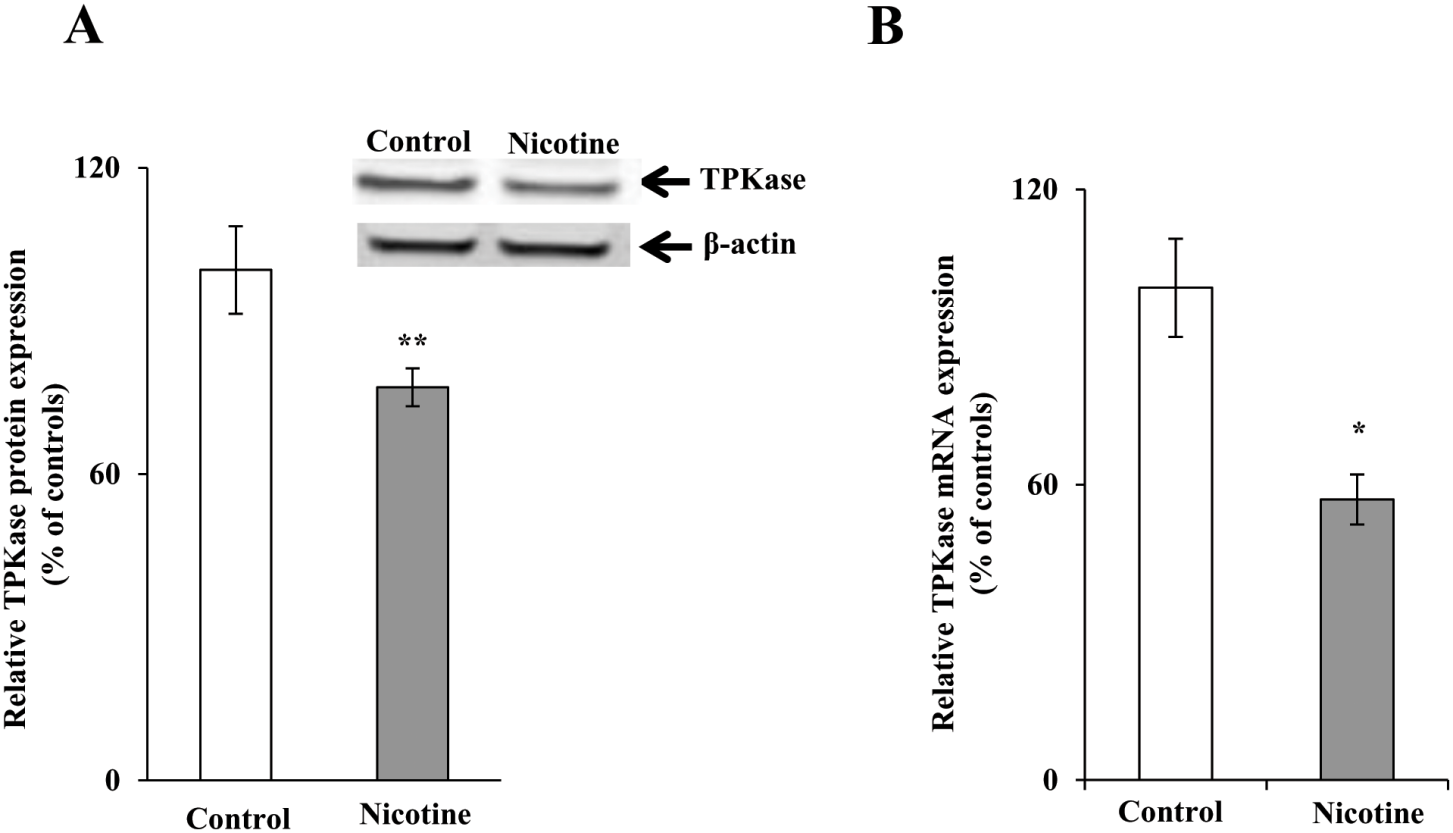
Chronic exposure of mouse primary PAC to nicotine reduces the levels of TPKase proteins (A) and mRNA (B). Western blot analysis was carried out using pancreatic acinar whole cell proteins (60 μg) isolated from chronic nicotine exposed mice and their controls. Real-time PCR was performed using mice gene-specific primers. Data are mean ± SE from separate sets of samples from multiple mice and were normalized relative to ARPO and calculated by the relative relationship method. *P < 0.01, **P < 0.05.

We also investigated the effect of chronic exposure of mice to nicotine on the level of expression of the key thiamin-metabolizing enzyme, thiamine pyrophosphokinase (TPKase) in PAC. The results showed that nicotine treatment causes a substantial reduction in the level of expression of TPKase protein and mRNA (*P <* 0.05 for protein and *P <* 0.01 for RNA) in PAC of mice exposed to nicotine chronically compared to their controls (Fig 4).

### Effect of chronic nicotine exposure on thiamin uptake by human PAC: *In vitro* exposure studies

**Fig 5:**
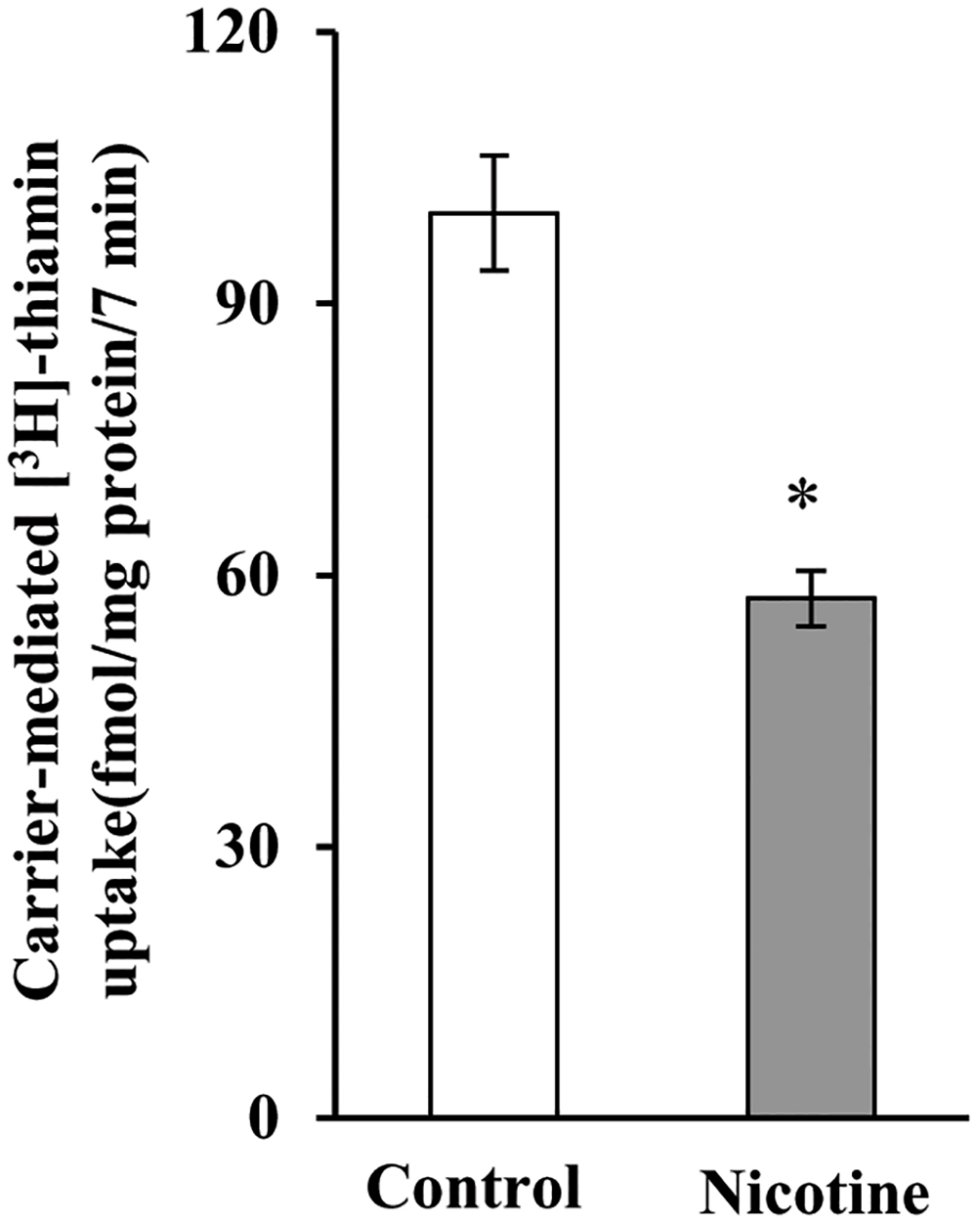
Chronic exposure of human PAC to nicotine decreases carrier-mediated [^3^H] thiamin uptake. Human PAC were exposed to nicotine (0.5 μM, 48 h) and carrier-mediated uptake of ^3^H-thiamin was determined as described under “Materials and Methods”. Data represents the mean ± SE of at least three separate uptake determinations.*P < 0.01.

**Fig 6:**
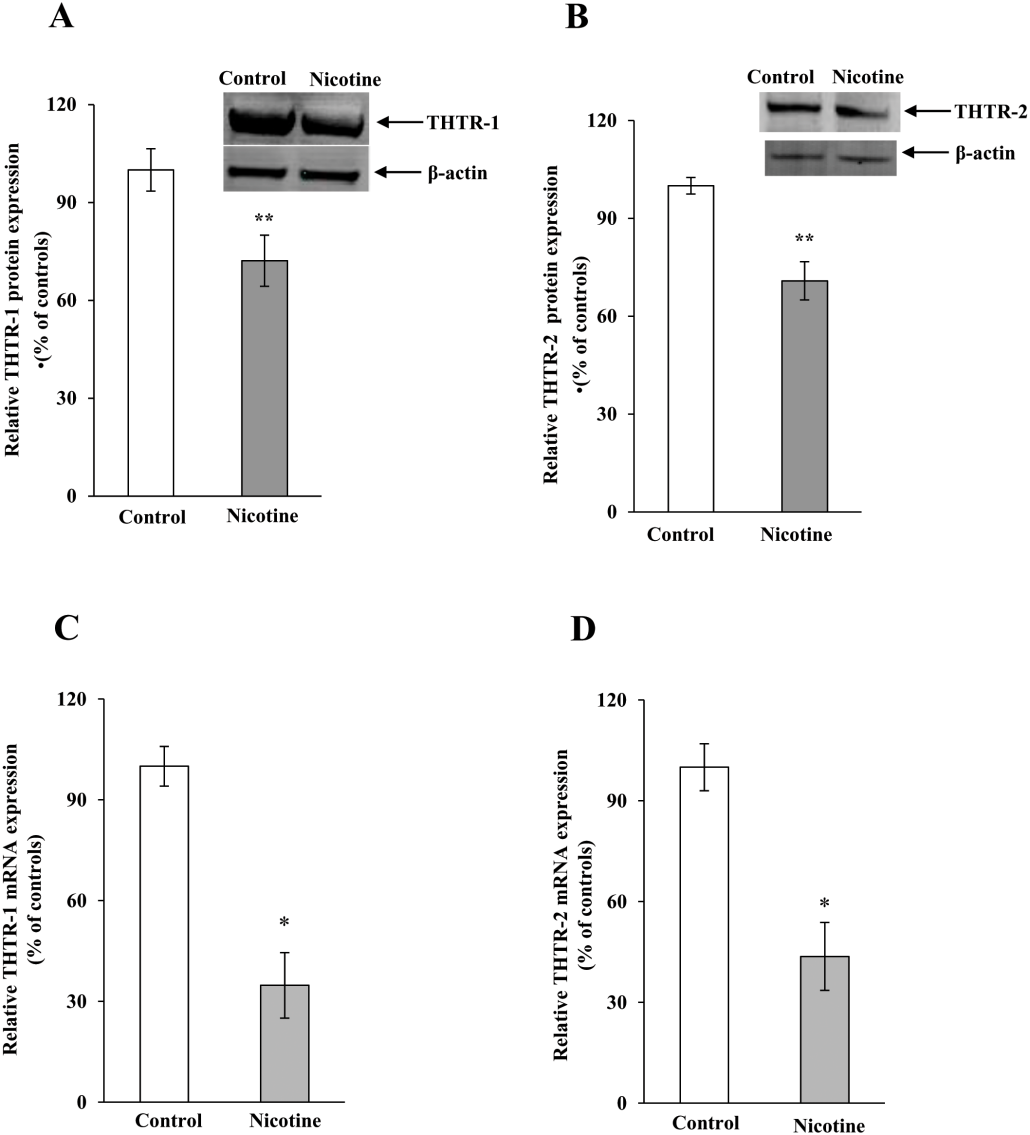
Chronic exposure of human PAC to nicotine reduces the levels of THTR-1 and THTR-2 proteins (A and B) and mRNA (C and D). Levels of THTR-1 and THTR-2 protein expression were determined by Western blotting. The mRNA expression was determined by quantitative PCR. Each data represents the mean ± SE of at least three independent experiments. *P < 0.01, **P < 0.05.

In this study, we used cultured human PAC that were isolated and maintained as described in “Methods”. We examined the effect of prolonged (48 h) exposure to nicotine [0.5 μM; a level similar to that found in pancreatic juice of smokers [48]] on the initial rate [5 min; [8, 9]] of carrier-mediated thiamin (15 nM) uptake. Treatment with nicotine was carried out as described [46, 47]. The total time human PAC were maintained *in vitro*; i. e., the time between their removal from the organ donors to their use in uptake investigations, was approximately 4 days. We used a recently described culture media [34] that is supplemented with STI, BSA and EGF to maintain human PAC. These factors appear to enhance PAC viability and maintain their differentiation, possibly by decreasing acinar cell injury by inhibiting proteases, including extracellular trypsin, released into the media (31). Also, we limited the time in culture to 3 days since previous studies [34] have shown that human PAC maintained under the above-described conditions for such a period undergo minimal morphological changes and do not transition to an epithelial phenotype (as shown by lack of epithelial surface markers in FACS analysis) [34]. Indeed, the level of mRNA expression of our two thiamin transporters, i. e., the *SLC19A2* and *SLC19A3* (measured by qPCR), were found to be the same on the day of isolation and on the day of use of the human PAC (relative quantity 1 ± 0.09 and 1 ± 0.08 for *SLC19A2* and *SLC19A3*, respectively). Also, the human PACs used in this study were able to secrete amylase on the day of use (measured by the Phadebas kit). Using these human PAC, our results showed nicotine exposure to lead to a significant (*P <* 0.01) inhibition in the initial rate of thiamin (15 nM) uptake (Fig 5). We also examined the effect of exposure of human PAC to nicotine on levels of expression of THTR-1 and THTR-2 proteins (Western blotting) and observed a significant (*P <* 0.05) reduction in the level of expression of both proteins in human PAC compared to untreated controls (Fig 6A and 6B). The effect of chronic exposure of human PAC to nicotine on the level of expression of THTR-1 and THTR-2 mRNAs was also tested by qPCR analysis. The results showed a substantial (*P <* 0.01) reduction in the expression of THTR-1 and THTR-2 mRNAs in human nicotine-exposed PAC cells compared to controls (Fig 6C and 6D).

## Discussion

Our aim in this study was to examine the effect of chronic exposure of PAC to a common environmental toxin, nicotine, on physiological and molecular parameters of thiamin uptake using *in vivo* and *in vitro* models. In the *in vivo* model we used transgenic mice carrying the human *SLC19A2* and *SLC19A3* promoters that we have previously established and characterized [32, 33]. We also examined freshly isolated PAC cells from mice exposed to nicotine and untreated controls for physiological/molecular investigations. In the *in vitro* model of chronic nicotine exposure, we used human PAC isolated from organ donors that were maintained in culture under optimal growth conditions, allowing these cells to maintain their viability and morphology but preventing their transformation to an epithelial phenotype (34). The human PAC cells used in these investigations exhibit similar expression levels of thiamin transporters up to four days after isolation, and thus represent a suitable model system for our studies.

Our results indicate that exposure of nicotine to transgenic mice carrying human *SLC19A2* and *SLC19A3* promoters fused to the reporter gene luciferase [32, 33] causes a significant inhibition in thiamin uptake by freshly isolated PAC and reduces the levels of thiamin transporters. The treatment also significantly reduced the level of expression of the mouse endogenous THTR-1 and THTR-2 proteins, mRNAs and hnRNA and was also linked to a marked reduction in the activity of the *SLC19A2* and *SLC19A3* promoters in the freshly isolated mouse PAC. The latter findings suggest that the effect of nicotine on thiamin uptake is mediated, at least in part, by a decrease in transcription of the *SLC19A2* and *SLC19A3* genes. Nicotine also negatively affects protein and mRNA expression of TPKase, a key metabolic enzyme involved in the rate-limiting step in thiamin metabolism. Collectively these results show that chronic exposure to nicotine decreases both the entry steps of thiamin into PAC cells and its subsequent intracellular processing.

Our results with the human PAC chronically exposed to nicotine *in vitro* showed that this exposure also decreases (by >40%) carrier-mediated thiamin uptake. Again the inhibition was found to be associated with a significant reduction of expression of THTR-1 and THTR-2 at the protein and mRNA levels. These results are similar to those observed with the mouse studies described earlier and validate the suitability of using human PAC as a model system.

In our review of the literature, it is apparent that earlier studies have been cautious regarding the use of cultured human PAC. However, the recent development of modified culturing conditions by Singh *et al* [34] and their demonstration that human PAC retain their viability, morphology and acinar phenotype (without transitioning towards epithelial phenotype) (see “Methods”) encouraged us to use these cells in our current studies. The fact that similar data was obtained with these cells and freshly isolated mouse primary PAC with response to the effects of nicotine on cellular thiamin transporters and metabolism provide further confirmation for the suitability of cultured human PAC in such type of investigations.

The effect of nicotine on thiamin uptake by PAC is similar to that seen previously with the nicotine metabolite, 4-(methylnitrosamino)-1-(3-pyridyl)-1-butanone (NNK) [51], a CS carcinogen that also accumulates in the pancreas [52]. This shows that multiple CS components can negatively impact pancreatic thiamin physiology and cellular homeostasis. The mechanism(s) that mediates the effect of nicotine on PAC thiamin uptake physiology is not clear at present but could be mediated by the nicotinic acetylcholine receptor [30]; further investigations are needed to address this issue. In conclusion, this study demonstrates for the first time that chronic exposure of PAC (both mice and human) to nicotine negatively impacts the physiological and molecular parameters of vitamin B1 uptake and that the effect is exerted, in part, at the level of transcription of the *slc19a2* and *slc19a3* genes. We anticipate that our future studies will find that low intracellular levels of thiamin could impair oxidative energy metabolism, increase oxidative stress and compromise mitochondrial structure and function [3]. The resulting decrease in cellular ATP level, might sensitize the pancreas to a secondary insult, predisposing it to development of pancreatitis and other pancreatic diseases [53].
